# Ideological values are parametrically associated with empathy neural response to vicarious suffering

**DOI:** 10.1093/scan/nsad029

**Published:** 2023-05-22

**Authors:** Niloufar Zebarjadi, Eliyahu Adler, Annika Kluge, Mikko Sams, Jonathan Levy

**Affiliations:** Department of Neuroscience and Biomedical Engineering, Aalto University, Espoo 02150, Finland; Department of Neuroscience and Biomedical Engineering, Aalto University, Espoo 02150, Finland; Department of Psychology, The Hebrew University of Jerusalem, Jerusalem 9190501, Israel; Department of Neuroscience and Biomedical Engineering, Aalto University, Espoo 02150, Finland; Department of Neuroscience and Biomedical Engineering, Aalto University, Espoo 02150, Finland; Aalto Studios—MAGICS, Aalto University, Espoo 02150, Finland; Department of Neuroscience and Biomedical Engineering, Aalto University, Espoo 02150, Finland; Baruch Ivcher School of Psychology, Reichman University, Herzliya 4610101, Israel

**Keywords:** neuropolitics, empathy, ideological asymmetry, alpha rhythm, magnetoencephalography

## Abstract

Several studies in political psychology reported higher levels of empathy among political leftists (i.e. liberals) as compared to political rightists (i.e. conservatives). Yet, all those studies lean on self-reports, which are often limited by subjective bias and conformity to social norms. Here, we tested this putative asymmetry using neuroimaging: we recorded oscillatory neural activity using magnetoencephalography while 55 participants completed a well-validated neuroimaging paradigm for empathy to vicarious suffering. The findings revealed a typical rhythmic alpha-band ‘empathy response’ in the temporal–parietal junction. This neural empathy response was significantly stronger in the leftist than in the rightist group. In addition to this dichotomous division, the neural response was parametrically associated with both self-reported political inclination and right-wing ideological values. This is the first study to reveal an asymmetry in the neural empathy response as a function of political ideology. The findings reported in this study are in line with the current literature in political psychology and provide a novel neural perspective to support the ideological asymmetry in empathy. This study opens new vistas for addressing questions in political psychology by using neuroimaging.

## Introduction

In recent years, there has been an increasing interest in investigating the connection between empathy and political ideology (Hasson *et al.*, 2018; Morris, 2020). Ideological values represent social and economic preferences, and recent research is trying to examine whether there is a psychological root such as the empathic ability for having particular values and preferences (Thorisdottir *et al.*, 2007). Psychological studies addressed this relation by asking subjects to rate their empathic reactions themselves (Hasson *et al.*, 2018). The purpose of the current research is to use neuroimaging as a quantitative unobstructive tool for evaluating empathic abilities to explore its association with political ideology. The left–right political spectrum is a system to describe citizens’ political ideology and determine their attitudes and approaches regarding the complex political issues in society (Thorisdottir *et al.*, 2007; Jost, 2021). For decades, researchers in social and psychological science have attempted to elucidate whether political inclination is rooted in psychological mechanisms and an individual’s personality traits or is affected by other external factors (Jost *et al.*, 2009; Brandt *et al.*, 2014a; Hasson *et al.*, 2018). On the one hand, extensive research stated political ideology as a reflection of psychological traits, and they specified characteristics of each ideological group based on the group values and goals (Jost *et al.*, 2003; Thorisdottir *et al.*, 2007; Morris, 2020). The leftist group (liberal) is usually addressed as a political group that supports egalitarian social policies, such as providing free education and health care for every member of society, and has more openness to social reforms to protect minorities and discriminated communities (Jost *et al.*, 2009; Morris, 2020). In contrast, the rightist group (conservative) has a greater desire to form and maintain hierarchical social structures and in-group unity and preserve social traditions (Alford *et al.*, 2005; Jost, 2006; Morris, 2020; Sidanius and Pratto, 2001). Besides, several recent studies indicated a greater level of political intolerance, attitude bias and feeling out-group threat for people who hold right-wing *vs* left-wing political beliefs (Lindner and Nosek, 2009; Stewart and Morris, 2021).

On the other hand, a contradictory view criticizes these attributions to leftists and rightists. They claim that there are more similarities between these two ideological groups than perceived before and their inclination toward social policy issues can be affected by the context and target group (Chambers *et al.*, 2013; Brandt *et al.*, 2014a). For instance, Brandt *et al.* (2014a) expressed that liberals and conservatives have a similar level of intolerance toward ideological opponents and threatening groups. They argued that inconsistent results in previous studies could be due to biased prompts, for example using a target group that was perceived to be more liberal for conservative participants (Brandt *et al.*, 2014a).

### Neuropolitics

Neuropolitics, an emerging field to study the interplay between neuroscience and politics, investigates brain mechanisms underlying complex political information processing such as political cognition and decision-making (Jost *et al.*, 2014; Schreiber, 2017; Haas *et al.*, 2020). Compared to commonly used surveys in political science studies, brain imaging measurements are more objective, more precise and less biased measurement techniques to evaluate questions in political psychology (Jost *et al.*, 2014). One of the main focuses of neuropolitical studies is to investigate whether there are any anatomical and functional brain differences among individuals with leftists and rightists political ideologies (Amodio *et al.*, 2007; Haas *et al.*, 2017, Schreiber, 2017; Haas et al., 2020).

To answer this question, previous studies mainly recorded and evaluated neural or functional responses in distinct brain regions involved in social and cognitive processing (Jost *et al.*, 2014; Haas *et al.*, 2020). For instance, several electroencephalography (EEG) and functional magnetic resonance imaging (fMRI) studies reported neurocognitive functional differences in the conflict-related anterior cingulate cortex (ACC) region between leftist and rightist groups (Amodio *et al.*, 2007; Weissflog *et al.*, 2013; Haas *et al.*, 2017). In EEG studies by Amodio et al. and Weissflog et al., the researchers detected a greater ACC activity during a Go/No-Go task in liberals *vs* conservatives, possibly reflecting the higher sensitivity for monitoring response conflict (Amodio *et al.*, 2007; Weissflog *et al.*, 2013). Weissflog et al. also found a positive correlation between ACC activity and preference for social equality, as well as an association between performance accuracy and openness to social change. Similarly, in an fMRI study by Haas and colleagues on the functional brain activity of liberals *vs* conservatives, regarding incongruent policy positions, the authors observed greater activations to incongruent trials in the insula and ACC in liberal participants (Haas *et al.*, 2017). These findings were in line with an MRI study that found structural differences and an increased ACC gray matter volume in liberals compared to conservatives (Kanai *et al.*, 2011). Another recent fMRI study on the neural underpinning of ideological differences in race categorization showed a positive correlation between anterior insula (AI) activity to racially ambiguous faces and conservatism (Krosch *et al.*, 2021).

### Empathy and political ideology

A growing body of literature on the association between empathy and political ideology suggests a higher general level of empathy in leftists *vs* rightists (Pratto *et al.*, 1994; Iyer *et al.*, 2012; Wagaman and Segal, 2014; Hasson *et al.*, 2018; McCue and Gopoian, 2000; Morris, 2020; Harell *et al.*, 2021). They suggested that the likely explanation for different empathic insights into the experience of others is the difference in their ground ideologies and policy preferences (Morrell, 2010; Wagaman and Segal, 2014). For instance, in a recent study, Hasson et al. used questionnaires to investigate the correlation between subjects’ political attitudes and (i) their motivation to feel empathy, (ii) their experienced empathy and (iii) their willingness to help others in three countries (Hasson *et al.*, 2018). They found that on average, liberals *vs* conservatives had more tendency to feel empathy and felt more empathy toward others, and in two out of three countries, they had more willingness to help others. This result was in line with a study by Pilskin et al. who found a higher desire in leftists *vs* rightists to support humanitarian policies (Pliskin *et al.*, 2014). In another study by Wagaman et al. on the relation of empathy and attitudes about government intervention concerning social welfare and well-being, results based on the questionnaires indicated a positive correlation between the general level of empathy and participants’ support for the government’s intervention in the above matters (Wagaman and Segal, 2014). They convincingly argue that stronger empathic insight motivates people to support egalitarian policies and contribute toward improving the welfare of others.

However, the association of empathy and political ideology has been typically limited to self-report questionnaires and surveys (Pratto *et al.*, 1994; Wagaman and Segal, 2014; Hasson *et al.*, 2018; Morris, 2020). To measure the participants’ empathic traits, these studies often utilized the interpersonal reactivity index (IRI) or similar behavioral scales and asked subjects to rate themselves their empathic ability by answering questions related to different aspects of empathy such as empathic concern (EC) or perspective-taking (PT). However, as indicated in several previous empathy studies, such evaluations sometimes have failed to precisely address participants’ empathic traits (Whitmarsh *et al.*, 2011; DiGirolamo *et al.*, 2019; Zebarjadi *et al.*, 2021). Besides, subjects might unconsciously or deliberately not report their true beliefs or preferences (Jost *et al.*, 2014; Hasson *et al.*, 2018). As discussed in the previous section, neuroimaging measurement offers a novel tool to study social and political psychology. In particular, it can be utilizable to investigate whether there is a correlation between political ideology and neural activity implicated in tasks involving empathy.

### Neuroscience of empathy

Understanding others’ emotional suffering and the ability to empathize with them is important for establishing friendships, making effective relationships and strengthening social bonds in society (Zaki, 2014). Empathy is a multifaceted psychological process with affective and cognitive components (Shamay-Tsoory *et al.*, 2009; Lamm *et al.*, 2011). Affective sharing is the ability to automatically mirror others’ emotions, and the cognitive facet is a complex top-down ability that emerges through mentalizing and taking others’ perspectives. The latter aspect is enabled by ‘putting oneself in others’ shoes’ and watching the world from their viewpoint. An fMRI study by Bruneau *et al.* (2012a) indicated two separate brain networks for empathy toward others’ emotional suffering, such as losing a close family member or divorce, and empathy toward others’ physical pain, such as hand or leg injuries (Bruneau *et al.*, 2012b). This study asserted that in empathy during emotional pain *vs* physical pain, the cognitive aspect of empathy is more prominent compared to sensory and affective aspects.

The empathy cognitive component, which involves thinking about others’ mental states, activates brain regions that majorly overlap with the theory of mind (ToM) brain networks (Völlm *et al.*, 2006; Bruneau *et al.*, 2012b). The overlapping areas mainly included temporal–parietal junction (TPJ) and medial prefrontal cortex regions (Saxe and Kanwisher, 2003; Völlm *et al.*, 2006; Van Overwalle, 2009; Bernhardt and Singer, 2012; Bruneau *et al.*, 2012b). Specifically, during social cognitive tasks, TPJ has been consistently recognized as the key area for understanding the emotional experiences of others and taking their perspective (Samson *et al.*, 2004; Perner *et al.*, 2006; Decety and Lamm, 2007; Van Overwalle, 2009; Cheon *et al.*, 2011; Saxe and Kanwisher, 2013; Schurz *et al.*, 2013). For instance, a lesion study by Samson et al. indicated that TPJ lesions result in specific ToM deficits and impairment of cognitive processes involved in social perception (Samson *et al.*, 2004). Besides, previous MEG studies showed that this temporoparietal and posterior temporal sulcus area is involved in social perception (Levy, Goldstein, Zagoory-Sharon, *et al.*, 2016) and predicts empathic interactions (Levy *et al.*, 2017). Finally, a meta-analysis by Schurz et al. selected TPJ as one of the main neural markers for PT and false-belief reasoning (Schurz *et al.*, 2013).

### Current study

**Fig. 1.**
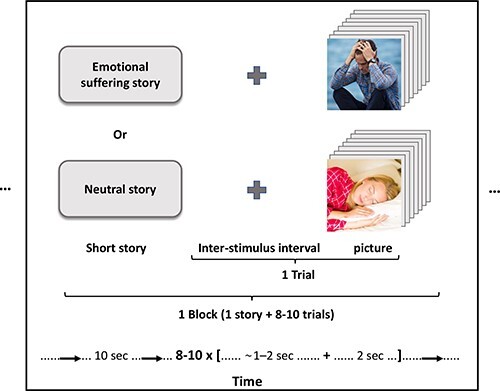
The experimental design of one block of the MEG paradigm. The experiment includes four emotional suffering blocks and four neutral blocks.

We use neuroimaging methods to investigate whether the brain response during empathy to vicarious emotional suffering confirms the results of the previous studies and reflects the self-reported differences between the two political groups. In addition, we aim to inspect whether this neural response is reflected in a region (TPJ) that captures the cognitive ability to empathize with others’ affective suffering. We record the subjects’ brain responses while implementing a typical empathy neuroimaging paradigm (Morelli *et al.*, 2014; Levy *et al.*, 2019a; Adi *et al.*, 2021), narrating short stories about protagonists followed by providing emotional suffering and neutral pictures of them to the participants (Figure 1). Given that we evaluate empathy in general in both political groups (and not intergroup empathy), we select here a target group that is not biased between the two ideologies.

Based on the prior psychological studies, we hypothesize that brain activation in the regions identified for empathy to others’ suffering (i.e. TPJ) will be different in the two ideological groups. We use MEG to capture frequency-decomposed neural activities and the source of the generated activities in the whole brain. Numerous brain imaging studies demonstrated the positive correlation between alpha-band power suppression and functional activation in a specified brain region (Pfurtscheller and Da Silva, 1999; Jensen and Mazaheri, 2010; Hanslmayr *et al.*, 2012; Schubring and Schupp, 2021). Besides, alpha-band activities were shown to be involved in plenty of cognitive, emotional and social tasks (Sadaghiani and Kleinschmidt, 2016; Schubring and Schupp, 2021), as well as in MEG empathy tasks (Whitmarsh *et al.*, 2011; Motoyama *et al.*, 2017; Levy *et al.*, 2018, 2019b; Zebarjadi *et al.*, 2021), and in this particular task used in Levy *et al.* (2019a). Therefore, to determine brain activation, we measure the statistical contrast of the alpha-band (∼10 Hz) power between the emotional suffering *vs* neutral (control) conditions. Increased alpha power suppression in the TPJ represents a greater brain activation in this region and a higher level of empathy. Based on the literature, we expect to observe more alpha suppression in the TPJ region in left-wingers compared to right-wingers, representing a higher level of empathy in the leftists’ group. In addition to the neural measurement, we evaluate participants’ political ideology and empathic ability using several self-report scales.

## Methods

### Subjects

Fifty-five healthy subjects (30 males and 25 females, mean age ± s.d. = 25.34 ± 3.87) were recruited via social media for a study investigating political attitudes and empathy. Participants were prescreened, and they were all MEG compatible with no serious neurological or psychiatric issues. The study received ethics approval from the IDC Herzliya ethics committee, and participants signed the consent form before the experiment.

### Experimental design

#### MEG paradigm

To measure brain responses during empathy toward others’ suffering, participants were exposed to the two contrasting conditions and they were asked to take the targets’ perspectives. Fourteen blocks of emotional suffering or control were randomly selected from a pool of seven suffering and seven control blocks and presented to the participants. The inter-block interval was jittered for ∼4–5 s. As illustrated in Figure 1, each block contained a contextual single-sentence story (which lasted for 10 s) followed by 8–10 related photos (each lasted for 2 s) with an inter-stimulus interval of ∼1–2 s. Sentences were designed to describe the situation in the ensuing photos and included *M* ± s.d. 9.07 ± 1.14 words and *M* ± s.d. 43.64 ± 5.10 long characters, with no statistically significant difference (*P* > 0.3) in the length between categories. Examples of sentences for suffering and neutral situations were ‘This woman just heard that there was a shooting in her son’s school’ and ‘This woman is ironing her clothes’, respectively. In total, there were 128 photos in uniform size of 300 × 225 pixels, half depicting vicarious emotional suffering and half depicting neutral situations (Figure 1). The physical parameters of the photos, such as complexity, contrast and luminance, were matched [no statistically significant difference (*P* > 0.35)], and photos’ affective valence and arousal differences between categories were assessed based on the ratings. Photos’ valence was rated as neutral (*M* ± s.d. 3.04 ± 0.25) and negative (*M* ± s.d. 1.95 ± 0.28) for the neutral and suffering stimuli, respectively [statistically significant difference between categories (*P* = 6.21 × 10^−47^)], and photos’ arousal was rated as low (*M* ± s.d. 2.05 ± 0.33) and high (*M* ± s.d. 3.83 ± 0.42) for the neutral and suffering stimuli, respectively [statistically significant difference between categories (*P* = 2.37 × 10^−53^)]. This paradigm was programmed and operated using E-Prime® 2 software (Psychology Software Tools Incorporated). The same paradigm has been previously used to investigate the neural mechanism of emotional empathy in the context of chronic trauma (Levy *et al.*, 2019a).

#### MEG recordings

Participants were laid in supine position inside the MEG scanner in a magnetically shielded room, and they were asked to not move their head or body during the measurement. To familiarize participants with the procedure, they watched two example blocks before the recording. Stimuli were presented in the center of a 20-inch monitor with a gray background at a viewing distance of 50 cm. The brain activity was recorded with a sampling rate of 1017 Hz (online 1–400 Hz band-pass filter) using a whole-head MEG with a 248-channel magnetometer array (4-D Neuroimaging, Magnes^®^ 3600 WH). The head position relative to the sensors was recorded using five coils attached to the subjects’ scalps. Additional reference coils above the subject’s head (∼30 cm) were utilized to cancel environmental noise.

#### Behavioral measures

##### Political ideology scale.

Participants’ general political ideology was self-reported using a seven-point political ideology scale ranging from 1 (extreme rightist) to 7 (extreme leftist). Further, we evaluated the subjects’ reading habits by asking them to rate their preferences regarding the daily leftist (Ha’aretz) and rightist (Israel Hayom) national-wide newspapers in Israel (Shultziner and Stukalin, 2021), from 1 (not at all) to 7 (all the time). The reading habit scales were utilized to check and validate the political ideology scale’s results. We categorized those who selected 1–3 on the political ideology scale as the rightist group and 5–7 as the leftist group (no inconsistency was found between political ideology and reading habit scales’ results). Out of 55 subjects, 25 were reported as leftists, eight as centrists (ones who rated 4 on the political ideology scale) and 22 as rightists. To divide the centrists, we categorized them according to their reading habits scale into rightist or leftist groups, so that five subjects were assigned to leftist and three subjects to rightist groups. Finally, 30 subjects for the leftist group and 25 subjects for the rightist group were considered and examined. It is important to note that none of the subjects rated their political ideology 1 or 7, which means that, there is no extreme rating in any of these groups. The average political ideology scores for the leftist and rightist groups are 5.3 and 2.5, respectively.

##### Right-wing authoritarian scale.

Subjects rated 10 items regarding authorization personality ranging from 1 (strongly object) to 6 (strongly agree) adopted from right-wing authoritarian (RWA) questionnaires (Altemeyer, 1983). This scale measured to what extent subjects respect and support traditional values endorsed by authorities. Referring to the previous studies, individuals with higher scores on this scale usually had more tendency toward right-wing political ideology (Manganelli Rattazzi *et al.*, 2007).

##### Empathic ability scale.

Subjects were asked to rate several items of EC and PT subscales in the IRI questionnaire from 1 (strongly disagree) to 7 (strongly agree).

##### Intergroup empathy feeling.

Participants rated their empathy feeling toward the opposite ideological group, (i.e. leftists toward rightists and rightists toward leftists) on a scale ranging from 1 (not at all) to 7 (to a great extent).

### Preprocessing and data analysis

Data preprocessing and analysis were done with MATLAB (MathWorks) and the FieldTrip software toolbox. Artifacts such as eye movement, eye blink and cardiac rhythm were removed using independent component analysis. Any remaining bad trials were visually inspected and rejected, and acceptable trials were band-pass filtered (1–40 Hz). Two out of 248 sensors were excluded due to malfunction. The onset of each trial is when the picture appears to the participants. To compute spectral power and Fourier transforms, epochs of 2500 ms (including a baseline period of 450 ms) were created on each trial, and a Hanning taper was applied to each time window with a sliding time window of 500 ms. As the oscillatory power includes both evoked and induced responses, subtracting evoked power components from oscillatory power resulted in induced activity generated in response to stimuli. Then, the power estimates for emotional suffering and neutral conditions were separately averaged across the tapered data epochs. Eventually, the statistical contrast between the two conditions was computed with a nonparametric statistical approach (Maris E, 2007), and a significant time–frequency window was detected.

To localize the source of the brain activities in all subjects, first, a single shell brain model was made using an Montreal Neurological Institute adult template brain. This model was adjusted for each subject using SPM8 (Wellcome Department of Imaging Neuroscience, University College London, www.fil.ion.ucl.ac.uk) to fit the manually digitized (Polhemus FASTRAK^®^ digitizer) head shape of the subject. Then, each subject’s brain volume was fractionated into a regular grid, and spatial filters for each grid location were reconstructed by a beamformer. These spatial filters that were based on the time–frequency window detected during sensor analysis only allowed activities from the location of interest. Subsequently, the brain activity patterns in this location of interest were evaluated on leftist and rightist groups separately, and virtual channel statistics were made on the beamformer window. During the statistical procedure, the *t*-values of the contrast between emotional suffering and neutral conditions for each participant were computed, and the group-level test statistic was assessed over the *t*-values. To find significant time–frequency clusters with random effects, each subject’s *t*-value was randomly multiplied by 1 or −1 to permute the original conditions and was summed over subjects. Randomization distribution was obtained by iterating this procedure 1000 times, each time evaluating the fraction of maximal and minimal cluster-level test statistic histogram for further computation to define the Monte Carlo significance probability.

## Results

**Fig. 2.**
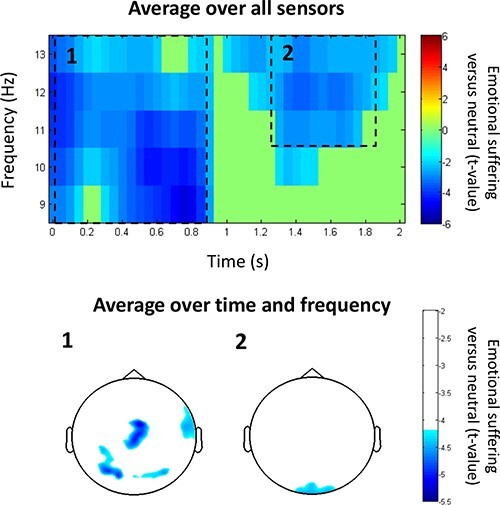
A time–frequency representation and topo plots of suppression patterns.

To compare the empathy level among people with different political ideologies, the brain response while observing and hearing others’ suffering (*vs* neutral) was recorded by MEG. Spectral analysis across all MEG sensors in the alpha-band frequency range and in the 0–2500 ms time window shows two main suppression patterns: (i) 0–850 ms in 9–13 Hz (*P*_cluster-cor_ = 9.9900e−004) and (ii) 1250–1850 ms in 11–13 Hz (*P*_cluster-cor_ = 0.0020). The time–frequency representation and topo plots are shown in Figure 2. Given that the earlier alpha suppression was more robust than the latter and that the earlier pattern was also discovered in the previous MEG study implementing the same empathy paradigm (Levy *et al.*, 2019a), we conducted further analysis only on this pattern.

**Fig. 3.**
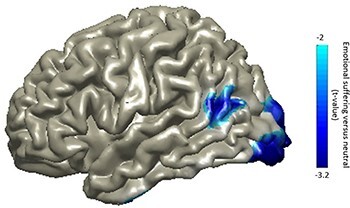
Localized activity in the alpha-band, depicted on an overlaid cortical surface.

The source of neural substrates was localized in the peak alpha frequency (11 ±2 Hz) and the time window of 0–850 ms. As it is represented in Figure 3, the alpha rhythm suppression was found to emanate from two significant cortical sources, with the peak values in the left TPJ (*P*_cluster-cor_ = 0.0190) extending to the posterior superior temporal sulcus (Beauchamp, 2015) and occipital (*P*_cluster-cor_ = 9.9900e−04) regions. Cognitive empathy (also known as ‘mentalizing’ or ‘theory of mind’) typically involves the activation of a set of regions in the brain, of which TPJ is one of the central components (Zaki and Ochsner, 2012; Marsh, 2018). We examined two coordinates in the left temporoparietal region reported in previous seminal studies investigating cognitive empathy/‘theory of mind’ with similar stimuli (Saxe and Kanwisher, 2003; Hynes *et al.*, 2006), and both coordinates were in the significant cluster that we detected here with the *t*-value of −3.2 and −2.4, respectively. This suggests that the TPJ activation reported here plausibly reflects cognitive empathy to vicarious distress. The occipital peak presumably was the typical occipital alpha suppression, related to perception and attention that was enhanced in the empathy-evoking condition (da Silva, 2013). Considering the cognitive empathy literature emphasizing the prominent role of TPJ (Zaki and Ochsner, 2012; Marsh, 2018), we continued further analysis on the TPJ source peak.

**Fig. 4.**
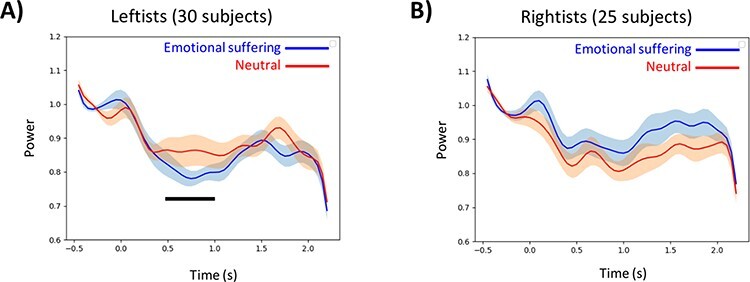
Alpha-band power temporal changes (normalized to baseline activity) in response to vicarious emotional suffering (blue line) and neutral (red line) stimuli in leftist (plot A) and rightist (plot B) political groups. The shades represent SEM, and the thick black line shows the statistically significant effect (P_cluster-cor_ < 0.001) on the time axis.

To evaluate the sources of alpha power activity changes in TPJ in the rightist and leftist groups, we proceeded with a virtual channel analysis in TPJ in the two groups, separately. Plots A and B in Figure 4 represent the temporal evolution of the alpha peak coordinate in response to others’ emotional suffering in the leftist and rightist groups, respectively. As illustrated in Figure 4A, the analysis revealed significant alpha suppression time points in the leftist group in the 450–1000 ms (*P*_cluster-cor_ = 0.0040) after stimuli onset. In contrast, no significant alpha suppression time point was observed in the rightist group (*P*_cluster-cor_ = 0.1608) (Figure 4B). This analysis confirmed what was found in the sensor analysis and the beamforming analysis, and it further indicated that the TPJ effect is rather a late effect, as it was not started at zero point. To further confirm the results, we replicated the virtual channel analysis on the two coordinates in the left temporoparietal region reported in previous studies with similar stimuli (Saxe and Kanwisher, 2003; Hynes *et al.*, 2006). Similarly, for these two coordinates, the results for the leftist group were significant, while for the rightist group were nonsignificant.

**Fig. 5.**
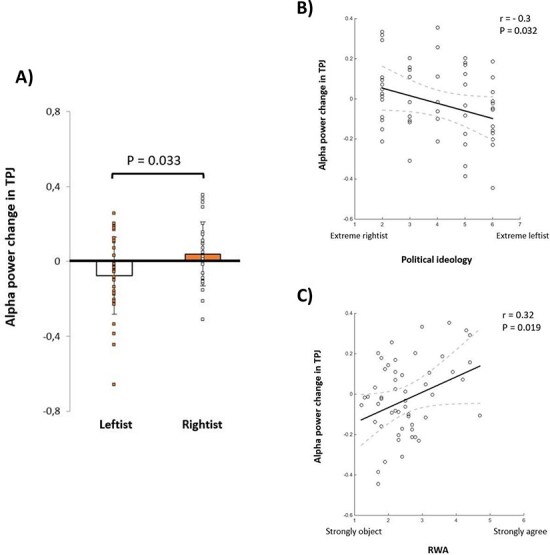
(A) Statistical contrast between averaged alpha power suppression in leftist and rightist groups (P = 0.033), (B) correlation between subjects’ political ideology and alpha power suppression in TPJ (r = −0.3, P = 0.032) and (C) correlation between subjects’ RWA scale and alpha power suppression in TPJ (r = 0.32, P = 0.019).

Subsequently, averaged alpha power changes in TPJ over the significant time window, selected from virtual channel analysis of all subjects, was calculated, and a statistical *t*-test between these values over subjects of the two political groups was conducted and provided in Figure 5A. Variations among leftist and rightist groups showed a significant statistical difference between these two ideological groups (*P* = 0.033). We also checked the effect while the centrist-leaning subjects were excluded, and the difference between the two groups was again significant (*P* = 0.048). This evidence states that the average power value difference between the two conditions in the leftist group is negative (*M* ± s.d. −0.076 ± 0.21), representing significantly robust alpha suppression in this group, while the average power value difference in the rightist group is positive (*M* ± s.d. 0.040 ± 0.17). In addition, to verify that the detected effect is an empathy-related activity, we conducted a similar analysis on the occipital source. For the occipital area, the statistical difference between the subjects of the two political groups was nonsignificant (*P*-value = 0.202), which means that the political ideology is particularly modulated empathy/ToM-related region.

To test whether the difference is dichotomous or parametric, we retested this alpha power against political ideology self-reported values. Figure 5B illustrates a correlation plot between alpha power changes in TPJ and subjects’ political ideology, indicating that the more inclination toward leftists’ ideology is associated with greater alpha power suppression in TPJ and a higher level of emotional empathy (*r* = −0.3, *P* = 0.032). Further, we tested whether this neural effect is associated with right-wing values in addition to self-reported political ideology. Figure 5C illustrates a correlation plot between alpha power changes in TPJ and subjects’ RWA scale responses, representing that the tendency for right-wing values explains the decrease in TPJ alpha power suppression and thereby reduced neural empathic response as captured in the current paradigm (*r* = −0.32, *P* = 0.019). The correlation between the political ideology scale and the RWA scale is moderately significant (*r* = −0.56, *P* < 0.001).

Finally, in addition to the neural empathy response, subjects rated EC and PT subscales in the IRI questionnaire, as well as their intergroup empathy feelings targeting the other ideological group, that is leftists toward rightists and rightists toward leftists. The analysis indicates that the difference in intergroup empathy feelings between leftists (*M* ± s.d. 3.66 ± 1.49) and rightists (*M* ± s.d. 3.24 ± 1.64) was not significant (*P* = 0.335). Likewise, no significant correlation between the self-reported EC and PT with neither TPJ effect (*r* = −0.001, *P* = 0.996 for EC and *r* = −0.140, *P* = 0.313 for PT) nor political ideology (*r* = −0.070, *P* = 0.615 for EC and *r* = 0.023, *P* = 0.866 for PT) was found.

## Discussion

The objective of the present study was to assess the association between political ideology and the general level of empathy to vicarious emotional suffering. Unlike the former studies that mainly evaluated this association through self-report surveys, we used MEG to measure brain response to empathy with others’ suffering and distress, in rightists and leftists. Our results indicated that the degree of empathy toward others’ suffering is reflected in the suppression of alpha rhythm generated in the TPJ region of the brain. This result is consistent with numerous previous studies (Samson *et al.*, 2004; Perner *et al.*, 2006; Whitmarsh *et al.*, 2011; Bernhardt and Singer, 2012; Bruneau *et al.*, 2012a; Schurz *et al.*, 2013; Motoyama *et al.*, 2017; Levy *et al.*, 2018, 2019b). Studies on TPJ indicated this brain region as one of the main areas involved in emotional and cognitive empathy, as well as mentalizing and PT. By dividing the subjects into left and right ideological groups, we observed a significantly stronger TPJ involvement among the leftist group compared to the rightist group. Greater TPJ activation among the leftists while listening and observing others’ suffering indicates that their neural empathic response, at least in the affective and cognitive context of this experiment, might be stronger than that of rightists. This unique neuroimaging finding further supports the results of several self-report studies that found a higher general level of empathy in the left *vs* right political group (Wagaman and Segal, 2014; Hasson *et al.*, 2018; Morris, 2020; Harell *et al.*, 2021). Importantly, our findings of parametric modulation of the neural empathic response are in line with the Graded Empathy hypothesis (Levy and Bader, 2020) and further emphasize the relevance of rhythmic measures (neural oscillations) of empathy (Zebarjadi *et al.*, 2021).

This discrepancy between rightists and leftists could be linked to the moral foundation and fundamental ideological differences between these two political groups. For instance, leftists’ greater tendency to support social and economic equality, their motivation to help low-status social groups and their low interest in social hierarchy can probably explain their stronger empathic abilities (Morris, 2020). These associations and the result of the correlation between the RWA scale and TPJ alpha power change accord with the earlier psychological research on the negative correlation between empathy and propensity for social hierarchy, which is typically attributed to rightist political views (Bäckström and Björklund, 2007; McFarland, 2010; Sidanius *et al.*, 2013). This negative relationship between the perception of suffering in others and the preference for social dominance hierarchy was also confirmed in an fMRI study (Chiao *et al.*, 2009). Chiao and colleagues recorded participants’ brain activity in response to observing painful *vs* neutral visual scenes. They found that the preference for hierarchical rather than egalitarian social relations, measured by the social dominance orientation scale, was negatively correlated with the functional activity in brain regions involved in pain empathy, such as AI and ACC.

To date, the majority of published studies on the correlation between empathy and political ideology have been based on subjective reports (Morris, 2020). Yet, using the self-report questionnaire as the only tool to evaluate participants’ empathy and its relation to political attitudes may not be adequate and carry out some bias (Jost *et al.*, 2014; Hasson *et al.*, 2018). The current study used both neuroimaging techniques as an objective measure and the IRI questionnaire as a self-report measure to assess participants’ empathy. Although we found a clear significant correlation between political ideology and empathy at the neural level, no evidence was found to reflect a relationship between political ideology and empathy levels as measured by IRI (i.e. EC, PT and intergroup empathy). A lack of neural–behavioral correlation was also reported in several previous studies (Whitmarsh *et al.*, 2011; DiGirolamo *et al.*, 2019; Zebarjadi *et al.*, 2021). One possible explanation might be the typical low sample size of the MEG studies (roughly 20 subjects) (Gross, 2019) compared to psychological studies that used exclusively self-report measures (roughly 300 subjects or more) (Wagaman and Segal, 2014; Hasson *et al.*, 2018; Harell *et al.*, 2021). Although this would require further inquiry, the current findings can be interpreted as indicative that empathy traits’ self-reports are most probably orthogonal to neural measures of empathy, which may be capturing empathy in a way that cannot be captured by self-reports.

The findings of this study can raise intriguing questions regarding the nature of empathy in humans and whether it might be systematically biased by political ideology. For instance, to the best of our knowledge, the neuroscience studies of empathy have overlooked political ideology as a dependent variable (Lamm *et al.*, 2011; Bernhardt and Singer, 2012). Since empathy is a complex multifaceted social ability, considering this association in future studies, in addition to other social circumstances and subjective experiences (Zebarjadi *et al.*, 2021), might lead to novel findings in this burgeoning field and provide deeper insights into the empathy phenomenon. Furthermore, to the best of our knowledge, this study is the first that uses MEG and reveals rhythmic neural activity in the field of neuropolitics. This novel neural perspective can open new vistas to neuropolitics studies (Schreiber, 2017).

A social psychological study suggested that there is no difference in the absolute level of empathic ability among opposite political groups and the found differences are due to the selected target groups (Waytz *et al.*, 2016). They argued that different cognitive–motivational styles, personality traits, motivational orientations and moral foundations among ideological groups and the way they view the social world can result in a greater tendency in conservatives to empathize with smaller, close and more well-defined social groups (e.g. family) and in liberals to empathize with larger and more permeable social groups. That study is also supported by the neural empathy intergroup bias that was measured in the Israeli context (Levy *et al.*, 2016a), although that study did not examine the possible contribution of political ideology. The present study was designed to evaluate empathy in general in both ideological groups, without any defined context or target group constrain. Therefore, the result needs to be interpreted with caution, since rightists’ *vs* leftists’ empathy toward a particular close target group (e.g. their family members), as indicated in Waytz’s study, has not been assessed. Furthermore, at the self-reported level, we assessed intergroup empathy levels (toward rightists *vs* leftists), and our results did not reveal any significant difference between the two groups, and rather moderate levels of empathy toward each other.

It is necessary to note that, first, similar to previous studies on this topic that consider the left–right dimension equivalent to the liberal–conservative dimension (Fuchs and Klingemann, 1990; Hasson *et al.*, 2018), throughout this paper, the terms leftist and liberal (and similarly, rightist and conservative) were used interchangeably. The liberal–conservative dimension is often used in the United States, whereas the left–right dimension is commonly used in Europe and Israel (Hasson *et al.*, 2018). Yet, it is important to bear in mind the social contexts in which these studies were conducted (Verkuyten, 2004). In the current study, ideological values strongly lean on the Israeli–Palestinian conflict: Bar-Tal and colleagues (2012) suggested a scale to measure individuals’ adherence to the ethos of conflict in Israel and demonstrated that this scale is negatively correlated with the support for peace-making policies. We used this scale in the current study, and the correlation between political ideology and ethos among participants was strong (*r* = −0.741, *P* < 0.001) and implied that right-wing ideology is strongly associated with the Israeli ethos. Therefore, the neuropolitical results reported in the present study can be specifically interpreted in the natural intergroup context wherein the study took place.

Second, this study aimed to investigate the possible association between political ideology and empathy toward vicarious suffering rather than searching for cause and effect. Therefore, it is important to take into consideration that it is not clear whether political ideology shapes empathy or empathy shapes political ideology. Third, it is important to note that the detected empathy level difference in the current study may not necessarily reflect empathic accuracy. However, the paradigm used here has been tested and validated in several previous studies, and it has been shown to effectively capture empathy (Morelli *et al.*, 2014; Levy *et al.*, 2019a, 2019b; Adi *et al.*, 2021).

Fourth, the present study emphasized on differences between the two ideological groups. Nonetheless, as discussed by Costello *et al.* (2022), there are also some commonalities and shared traits in rightist and leftist individuals with extreme ideological views (Brandt *et al.*, 2014b; Costello *et al.*, 2022). For instance, both groups tend toward social uniformity and have prejudice, cognitive rigidity and aggression toward the rival group. Similarly, some other studies indicated intolerance in both right and left political groups toward people with opposing beliefs, values and attitudes (Crawford and Pilanski, 2014; Brandt *et al.*, 2014b). Moreover, a study by Pilskin et al. searched for the interactive influence of ideology and emotional processes and found that leftists’ policy support (compared to rightists) is more related to their positive and negative emotions (Pliskin *et al.*, 2014). They argued that the high influence of the emotional process on leftists might guide their policy support and lead to intermittent political positions rather than stable and long-term ones. Considering all these points, it is crucial not to overlook the leftists’ prejudice, intolerance or other negative attitudes toward the rightists as well as their weaknesses in taking stable political positions.

## Conclusion

The paradigm used in the current study investigated empathy toward other individuals’ emotional suffering and misfortunes. The results confirmed a typical empathy response in alpha rhythm in the brain’s TPJ. The neural response was significantly stronger in the leftist *vs* rightist group and was parametrically modulated by political inclination and driven by right-wing values. Yet, one cannot exclude the possibility that the brain of rightists might respond differently, depending on other empathic contexts. However, our study further supports the observation that leftists *vs* rightists might respond more empathetically to others’ suffering.

## Data Availability

The data underlying this article can be shared on reasonable request to the corresponding author, pending institutional approval.
